# Supplementary material for: Ampicillin-susceptible Enterococcus faecium infections: clinical features, causal clades, and contribution of MALDI-TOF to early detection
Source: Microbiol Spectr. 2023 Sep 25;11(5):e04545-22. doi: 10.1128/spectrum.04545-22 (PMC10581188; doi:10.1128/spectrum.04545-22)
Supplement: Figure S1 — Genetic environment of pbp5 and distribution of isolates according to clades and ampicillin susceptibility. [file spectrum.04545-22-s0001.pdf]

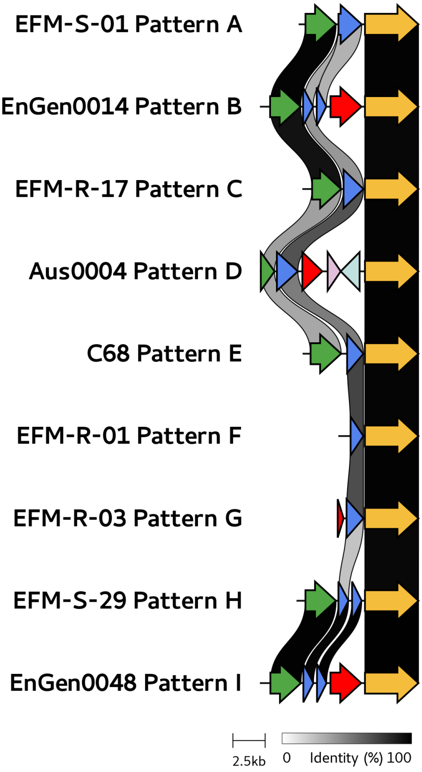

ftsW msr pbp5 IS gnat hisJ

	Number of genomes				
	Clades*			Ampicillin **	
	A1	A2	B	S	R
EFM-S-01 Pattern A	1	32	20	50	1***
EnGen0014 Pattern B	0	5	0	4	1
EFM-R-17 Pattern C	12	4	0	0	16
Aus0004 Pattern D	7	0	0	0	7
C68 Pattern E	1	0	0	0	1
EFM-R-01 Pattern F	15	0	0	15	0
EFM-R-03 Pattern G	8	0	0	8	0
EFM-S-29 Pattern H	1	6	0	0	6
EnGen0048 Pattern I	0	1	0	0	1

Figure S1. Genetic environment of *pbp5* and distribution of isolates according to clades and ampicillin susceptibility. The physical maps represent the five patterns described by Montealegre *et al.* (A to E) and the four newly described patterns (F to I). The name of a representative strain is shown for each pattern. Genes and insertion sequences are indicated by arrows of different colors. The links between physical maps are coloured according to the protein identity between sequences. *The hybrid genomes EnGen0002 and 1_231_408 were not considered. **Isolates without ampicillin susceptibility data were ignored. ***The isolate EnGen0052 was considered as pattern A based on whole genome sequence analysis.
