# Supplementary material for: Ampicillin-susceptible Enterococcus faecium infections: clinical features, causal clades, and contribution of MALDI-TOF to early detection
Source: Microbiol Spectr. 2023 Sep 25;11(5):e04545-22. doi: 10.1128/spectrum.04545-22 (PMC10581188; doi:10.1128/spectrum.04545-22)

Figure S2. Coregenome-based phylogenetic tree of the 52 isolates from our study and the 72 isolates from the study of Lebreton *et al.* The clades A1, A2 and B are highlighted in salmon, purple and blue, respectively. The ampicillin susceptibility of each isolate, determined by disk diffusion or from the literature, is indicated by a circle and a triangle, respectively. Grey circles represent missing values. The pbp5 environment pattern, the insertion after position 466 and the residu at position 485 of PBP5 are indicated for each isolate. Where the pattern and/or mutations could not be determined, they are indicated by "NA". The scale represents genetic distances in nucleotide substitutions per site.

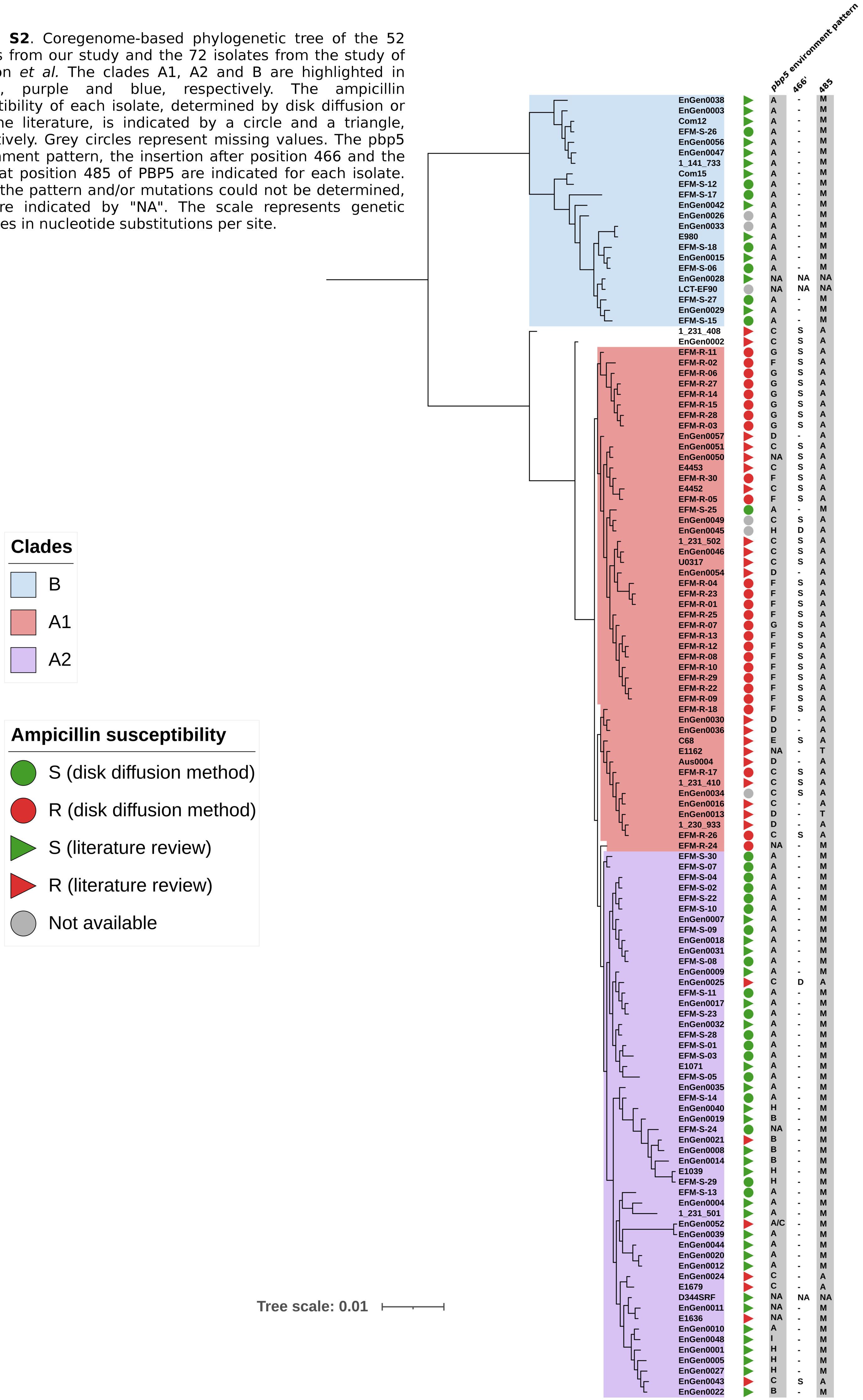

Supplement: Figure S2 — Coregenome-based phylogenetic tree of the 52 isolates from our study and the 72 isolates from the study by Lebreton et al. [file spectrum.04545-22-s0002.pdf]
