# Supplementary material for: Ampicillin-susceptible Enterococcus faecium infections: clinical features, causal clades, and contribution of MALDI-TOF to early detection
Source: Microbiol Spectr. 2023 Sep 25;11(5):e04545-22. doi: 10.1128/spectrum.04545-22 (PMC10581188; doi:10.1128/spectrum.04545-22)
Supplement: Figure S3 — Dendrogram calculated from the Mass Spectra Profiles (MSP) of the 27 isolates used to construct the MALDI-TOF MS database. [file spectrum.04545-22-s0003.pdf]

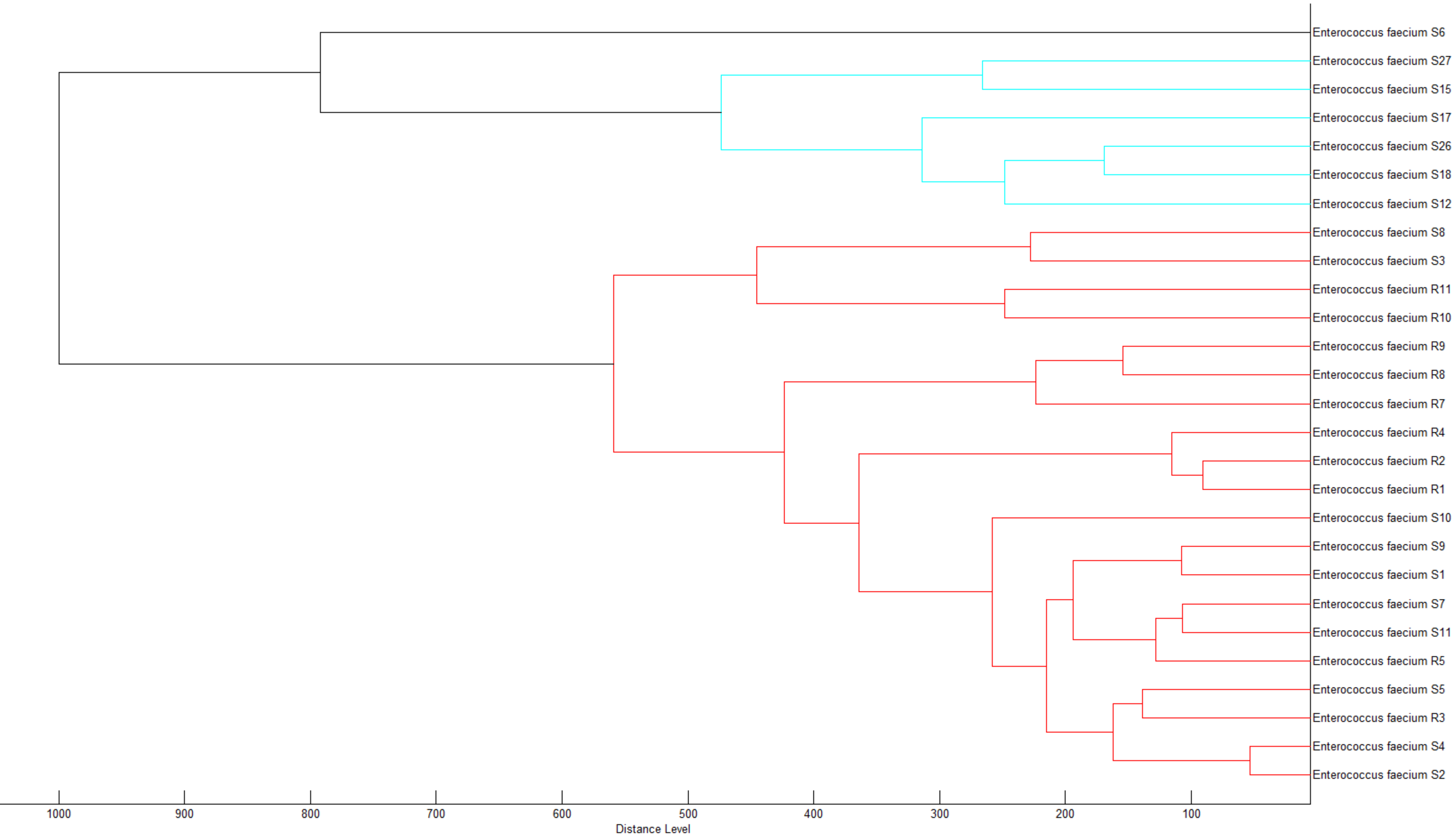

Figure S3. Dendrogram calculated from the Mass Spectra Profiles (MSP) of the 27 isolates used to construct the MALDI-TOF MS database. The dendrogram was constructed with the MALDI BioTyper compass explorer v4.1, with the distance set to 'Euclidean' and the linkage set to 'complete'. Isolates from clade B are shown with branches in blue, except for the divergent isolate EFM-S-06 in black. Isolates from the A1/A2 clade are shown with branches in red.
