# Supplementary material for: Ampicillin-susceptible Enterococcus faecium infections: clinical features, causal clades, and contribution of MALDI-TOF to early detection
Source: Microbiol Spectr. 2023 Sep 25;11(5):e04545-22. doi: 10.1128/spectrum.04545-22 (PMC10581188; doi:10.1128/spectrum.04545-22)
Supplement: Figure S4 — Example of mass spectra ranges obtained from the 27 isolates used to build the MALDI-TOF MS database [file spectrum.04545-22-s0004.pdf]

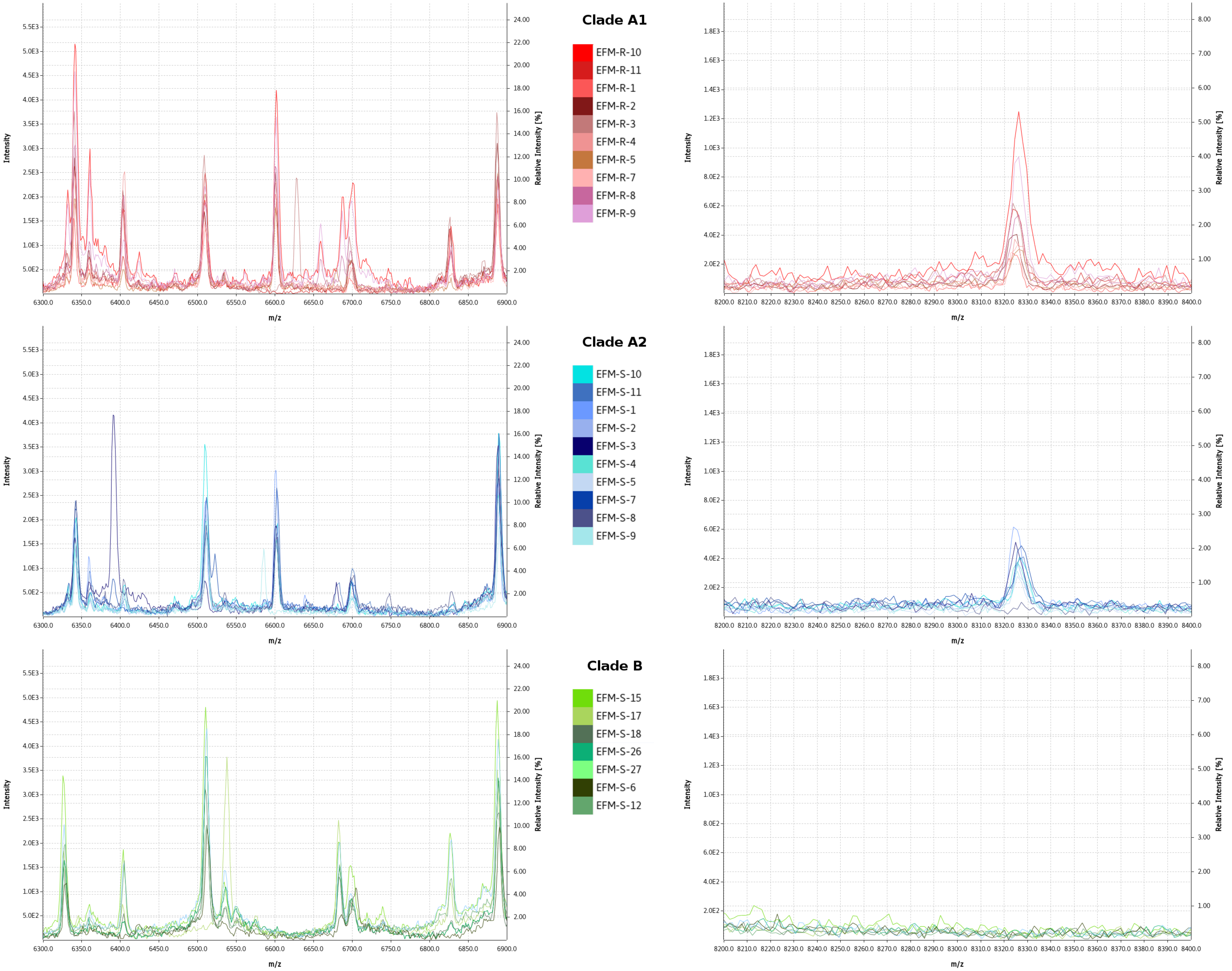

Figure S4. Example of mass spectra ranges obtained from the 27 isolates used to build the MALDI-TOF MS database. A) m/z ranges from 6300 to 6900. B)m/z ranges from 8200 to 8400. Isolates from the A1 clade are shown in red, those from the A2 clade in blue and those from the B clade in green. Spectra were read with the open source software OpenChrom v1.5.0 (http://www.openchrom.net/).
